# A causal inference framework to compare the effectiveness of life‐sustaining ICU therapies—using the example of cancer patients with sepsis

**DOI:** 10.1002/ijc.70138

**Published:** 2025-09-08

**Authors:** João Matos, Tristan Struja, Naira Link Woite, David Restrepo, Andre Kurepa Waschka, Leo A. Celi, Christopher M. Sauer

**Affiliations:** ^1^ Faculty of Engineering University of Porto Porto Portugal; ^2^ Laboratory for Computational Physiology, Institute for Medical Engineering and Science Massachusetts Institute of Technology Cambridge Massachusetts USA; ^3^ Medical University Clinic Kantonsspital Aarau Aarau Switzerland; ^4^ Clinic of Internal Medicine Spital Muri Muri Switzerland; ^5^ Telematics Department University of Cauca Popayan Colombia; ^6^ College of Liberal Arts and Sciences Mercer University Macon Georgia USA; ^7^ Mathematics & Statistics Elon University Elon North Carolina USA; ^8^ Department of Hematology & Stem Cell Transplantation, West German Cancer Institute University Hospital Essen Essen Germany; ^9^ Institute for Artificial Intelligence in Medicine University Hospital Essen Essen Germany

**Keywords:** cancer, critical care, eICU‐CRD, health equity, MIMIC‐IV, sepsis, TMLE

## Abstract

The rise in cancer patients could lead to an increase in intensive care units (ICUs) admissions. We explored differences in treatment practices and outcomes of invasive therapies between patients with sepsis with and without cancer. Adults from 2008 to 2019 admitted to the ICU for sepsis were extracted from the databases MIMIC‐IV and eICU‐CRD. Using Extreme Gradient Boosting, we estimated the odds for invasive mechanical ventilation (IMV) or vasopressors. Targeted maximum likelihood estimation (TMLE) models estimated treatment effects of IMV and vasopressors on in‐hospital mortality and 28 hospital‐free days. 58,988 adult septic patients were included, of which 6145 had cancer. In‐hospital mortality was higher for cancer patients (30.3% vs. 16.1%). Patients with cancer had lower odds of receiving IMV (aOR [95%CI], 0.94 [0.90–0.97]); pronounced for hematologic patients (aOR 0.89 [0.84–0.93]). Odds for vasopressors were also lower for hematologic patients (aOR 0.89 [0.84–0.94]). TMLE models found IMV to be overall associated with higher in‐hospital mortality for solid and hematological patients (ATE 3% [1%–5%], 6% [3%–9%], respectively), while vasopressors were associated with higher in‐hospital mortality for patients with solid and metastatic cancer (ATE 6% [4%–8%], 3% [1%–6%], respectively). We utilized US‐wide ICU data to estimate a relationship between mortality and the use of common therapies. With the exception of hematologic patients being less likely to receive IMV, we did not find differential treatment patterns. We did not demonstrate an average survival benefit for therapies, underscoring the need for a more granular analysis to identify subgroups who benefit from these interventions.

## Graphical abstract

What's new?

Rising cancer incidence in the United States is associated with an increased demand on intensive care units (ICUs). Critically ill cancer patients, however, often rely on life‐sustaining therapies, which are linked to greater ICU mortality. Here, the authors estimated causal inference between life‐sustaining therapies and in‐hospital mortality in ICU patients with sepsis and cancer. Critical cancer patients with sepsis were as likely to receive invasive mechanical ventilation and vasopressors as patients with sepsis alone. Survival benefits for invasive therapies were not observed, indicating a need for further investigation to identify patient subgroups who are best served by these interventions.

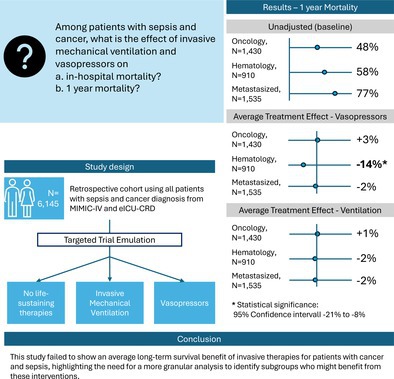

**Abbreviations**
APACHE: acute physiology and chronic health evaluation
ATE: average treatment effect
BIDMC: Beth Israel Deaconess Medical Center
CCI: Charlson comorbidity index
CI: confidence intervals
ECMO: extracorporeal membrane oxygenation
eICU‐CRD: eICU Collaborative Research Database
ICD‐10: International Classification of Diseases, Tenth Revision
ICU: intensive care unit
IMV: invasive mechanical ventilation
LOS: length of stay
MIMIC‐IV: Medical Information Mart for Intensive Care IV
OASIS: Oxford Acute Severity of Illness Scores
OR: odds ratios
PMP: predicted mortality probability
STROBE: strengthening the reporting of observational studies in epidemiology
TMLE: targeted maximum likelihood estimation
TNM: tumor node metastasis staging system

## 1 INTRODUCTION

Advances in the diagnosis and treatment of cancer have resulted in an increased length of survival, resulting in an increased likelihood of intensive care unit (ICU) admissions. One in 20 patients with cancer is admitted to the ICU within 2 years of cancer diagnosis.^1^, ^2^ Additionally, one in seven to one in 10 ICU patients carries a cancer diagnosis.^3^, ^4^ Patients with cancer are a heterogenous group in terms of disease sites and pathology, with large variations in short‐and long‐term overall and cancer‐specific survival.^4^, ^5^ Furthermore, ICU survival in general is also highly dependent on local admission policies, which vary significantly between institutions and countries.^6^

Thus, it is important to understand factors contributing to differences in outcomes of critically ill patients with cancer. Some studies have found no difference in in‐hospital mortality between ICU patients with and without cancer.^7^ In other studies, patients with hematological cancer had two‐fold elevated in‐hospital mortality rates compared to those with solid tumors or no cancer at all.^4^, ^8^ Furthermore, common life‐sustaining therapies such as invasive mechanical ventilation (IMV) and use of vasopressors have been associated with significantly increased ICU mortality for patients with cancer.^3^, ^7^, ^9^

We aimed to estimate a causal inference between the life‐sustaining therapies IMV and vasopressors with in‐hospital mortality and 28 hospital‐free days in patients with sepsis and cancer using the causal inference framework and target trial concept proposed by Hernan and others.^10^, ^11^ This framework allows for the use of the wording of causality in retrospective studies.^12^ Moreover, we aimed to identify patient subgroups benefitting least from life‐sustaining therapies. We believe this could aid physicians with clinical decision making for this heterogeneous and clinically challenging patient group. Randomized controlled trials may be unethical in this context, as IMV and vasopressors are established therapies with proven benefit in specific subgroups. Critically ill cancer patients represent a particularly vulnerable population due to frequent comorbidities, cumulative organ damage from malignancy and treatment, advanced age, and often limited life expectancy. These factors make them ideal candidates for causal inference methodologies, which allow for robust analysis without withholding standard‐of‐care interventions.

## 2 METHODS

The results of this study are reported in accordance with the Strengthening the Reporting of Observational studies in Epidemiology (STROBE) statement.^13^ We used two ICU datasets to conduct our retrospective cohort study: the Medical Information Mart for Intensive Care IV (MIMIC‐IV) v2.2 database^14^ and the eICU Collaborative Research Database (eICU‐CRD).^15^ MIMIC‐IV is a publicly available database that contains information from real ICU stays of patients admitted to one tertiary academic medical center, Beth Israel Deaconess Medical Center (BIDMC), in Boston, United States between 2008 and 2019. The eICU data does not encompass MIMIC‐IV data. Both databases contain comprehensive information from ICU stays including vital signs, laboratory measurements, medications, and mortality data. Data were last accessed November 30, 2023.

We hypothesized that treatment allocation of ICU interventions is not equally distributed across cancer status, leading to differences in outcomes. We suspected that patients with cancer and sepsis experienced a more harmful use of ICU interventions, especially in less severely ill patients, and that these effects would vary across illness severity, as gauged by the Oxford Acute Severity of Illness Scores (OASIS) and APACHE scores.

### 2.1 Outcome definition

The primary outcome was in‐hospital mortality, including discharge to hospice care. The secondary outcome was 28‐hospital free days. One‐year mortality was chosen as a secondary outcome for MIMIC cases only, as follow‐up data is not available in eICU. Consistent with recent research, we employed the timing of discharge or death at an odd versus an even hour as a negative control outcome, as it should not be affected by treatment.

### 2.2 Target trial definition

All patients included in the analysis were older than 18 years and had sepsis as defined by the sepsis–3 criteria.^16^ Only first‐time ICU stays were included, and cases with missing discharge location were excluded. Patients were also excluded if their length of stay was less than 1 day to ensure a homogenous cohort. To mitigate immortal time bias, treatment assignment for IMV (excluding non‐invasive ventilation) and vasopressors was limited to an eligibility period within the first 24 h. If treatment was started after this eligibility period, the patient was retained in the control group, generating a target trial to avoid immortal time bias.^17^ All decisions on data extraction and selection followed the causal inference and target trial concepts and therefore findings are described using causal language^10^, ^11^ in case the following assumptions are satisfied: (1) Ignorability assumption: after adjusting for confounders as ascertained by domain experts, treatment allocation should be random; (2) Positivity assumption: across all strata, both the intervention and control have been received; (3) Consistency assumption: no interference between units and a constant treatment, a reasonable assumption in most clinical questions.^18^ We summarized our decision in the appendix as proposed by experts in the field (see Supplementary Table 1).^19^, ^20^

### 2.3 Cancer ICD‐10 Codes

We used International Classification of Diseases, Tenth Revision (ICD‐10)^21^ codes to search for cancer diagnoses in a patient stay. Mapping between ICD‐10 codes and cancer types was done by expert knowledge and is provided in the supplement (see Supplementary Table 2). We used a publicly available crosswalk to map the ICD‐10 codes to ICD‐9 codes for earlier stays in MIMIC‐IV.^22^ The cancer diagnoses were then grouped into either solid cancer, metastasized cancer, or hematologic cancer (see Supplementary Table 3). Cancer types were hierarchically categorized, that is, metastasized would forgo hematological cancer, which would forgo solid cancer in patients with multiple diagnoses of cancer.

### 2.4 Covariate definition

Data on time‐varying covariates were ignored if recorded before the time of ICU admission; the remaining data was aggregated for the first 24 h of the stay by taking the maximum, minimum, or mean value as appropriate, and 49 pertinent variables were used to adjust for confounding (see Supplementary Table 4 and supplementary Figure 1). First and last documented resuscitation orders were extracted to control for vigor of care. We also retrieved ICD‐10 codes for key comorbidities, the OASIS in MIMIC‐IV, and APACHE IVa scores in eICU‐CRD. To obtain a common ground truth, we used the predictions formulas of the OASIS and APACHE scores to calculate a predicted mortality probability (PMP). Patients were placed in PMP tertiles as having low, moderate, or high sepsis severity.

### 2.5 Statistical analysis

Statistical analysis was performed using R version 4.2^23^ and Python 3.10.9^24^ running on Visual Studio Code. For summary statistics, we used the table1 R package.^25^ To gauge the likelihood to receive one of the three interventions stratified by cancer type where patients without a cancer diagnosis served as the reference, we fitted a nonlinear XGBoost model^26^ reporting our findings as odds ratios with 95% confidence intervals (OR 95% CI) based on SHAP‐values.^27^, ^28^ The confidence intervals were computed with 100 iterations of estimation with a 5‐fold train/estimation procedure. At each iteration and for each fold, 20% of the data was used to train the model and compute the interval before averaging on the 5 folds. The final OR is the median of all iterations. This procedure is mimicking the computation of OR in logistic regression using bootstrapping.

To draw causal inferences, we fitted targeted maximum likelihood estimation (TMLE) models to compute the average treatment effect (ATE) for each intervention, stratified by cancer type and PMP. TMLE is a semiparametric framework estimating the causal effect of an intervention. We used the integrated SuperLearner package, an ensemble machine learning algorithm with 5‐fold cross‐validation to model the counterfactual outcome. SuperLearner weighs results from multiple statistical algorithms to create a best performing prediction. These stacked and weighed predictions are statistically expected to perform at least as good as a single optimal prediction algorithm. We used the following Superlearners: SL.mean—simple mean prediction, SL.glm—generalized linear models, SL.glmnet—generalized linear models penalized using elastic net, SL.ranger—random forest models, and SL.earth—multivariate adaptive regression spline models. For a graphical depiction of the sequence of methodology used, please refer to supplementary Figure 2.

Assignment to each intervention was assumed to be independent of the outcome, thereby ensuring conditional exchangeability. There were no multiple versions of a treatment, as we dichotomized all treatments, thus upholding the consistency assumption. We tabulated the interventions according to cancer type and PMP to check if all strata had a non‐null frequency of an intervention, a way of checking for violations of the positivity assumption.^29^, ^30^ We consider our work hypothesis generating, which is why we abstained from computing p‐values and only provide 95% confidence intervals.

## 3 RESULTS

**FIGURE 1:**
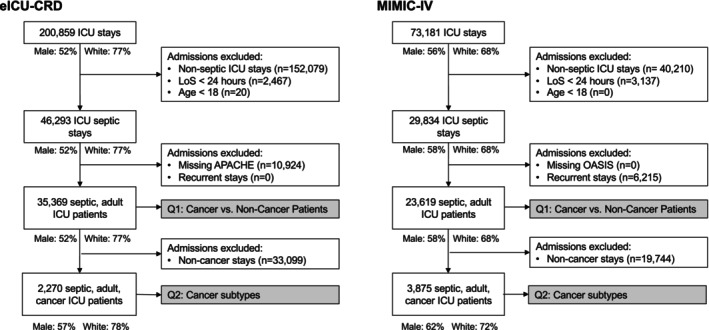
Study cohort selection flow chart, in eICU‐CRD (left), and MIMIC‐IV (right). APACHE, Acute Physiology and Chronic Health Evaluation score; ICU, intensive care unit; OASIS, Oxford Acute Severity of Illness Score; Q1, research question 1; Q2, research question 2; LoS, length of stay (in the ICU).

After applying our inclusion and exclusion criteria on both the MIMIC‐IV and eICU‐CRD datasets, 58,988 patients were retained, of which 23,619 were derived from MIMIC and 35,369 from the eICU‐CRD database. Among these patients, 6145 had a cancer diagnosis, 3875 from MIMIC‐IV and 2270 from eICU‐CRD (see Figure 1). Colorectal, lung, non‐Hodgkin lymphoma, and leukemia were the most common cancers (see Supplementary Table 2). Median age was 67 years among all categories, 45.6% of our cohort were female, and 73.6% were White (see Table 1). Among patients with a cancer diagnosis, in‐hospital mortality was 30.3%, compared with 14.5% for non‐cancer patients. Baseline vasopressors use, SOFA score, full code on admission and discharge, hypertension, and COPD were balanced between groups. On the other hand, there were marked differences in IMV use (39.8% vs. 48.2%, respectively); median Charlson comorbidity index (CCI, excluding cancer diagnosis, 8 vs. 5, respectively) was comparable between the groups when accounting for the two points attributed to the cancer diagnosis, while the prevalence of heart failure, asthma, and chronic kidney disease were all lower in the cancer group.

**TABLE 1:** Baseline information on the first study cohorts, with cancer and non‐cancer patients, after merging MIMIC‐IV and eICU‐CRD.

	Cancer (*N* = 6,145)	Non‐Cancer (*N* = 52,843)	Overall (*N* = 58,988)
In‐hospital mortality	1,864 (30.3%)	7,656 (14.5%)	9,520 (16.1%)
Length of stay if died (days) Median (Q1, Q3)	4.13 (2.21, 8.13)	5.17 (2.58, 10.3)	4.96 (2.50, 9.84)
Length of stay if survived (days) Median (Q1, Q3)	4.21 (2.21, 8.41)	5.46 (2.92, 9.84)	5.33 (2.85, 9.75)
Invasive mechanical ventilation	2,446 (39.8%)	25,480 (48.2%)	27,926 (47.3%)
Vasopressor(s)	2,530 (41.2%)	22,591 (42.8%)	25,121 (42.6%)
Age overall (years) Median (Q1, Q3)	67.0 (59.0, 76.0)	67.0 (55.0, 78.0)	67.0 (55.0, 78.0)
Sex (female)	2,517 (41.0%)	24,389 (46.2%)	26,906 (45.6%)
Race			
Asian	198 (3.2%)	942 (1.8%)	1,140 (1.9%)
Black	581 (9.5%)	5,355 (10.1%)	5,936 (10.1%)
Hispanic	262 (4.3%)	2,210 (4.2%)	2,472 (4.2%)
Other	559 (9.1%)	5,444 (10.3%)	6,003 (10.2%)
White	4,545 (74.0%)	38,892 (73.6%)	43,437 (73.6%)
SOFA Median (Q1, Q3)	5.00 (3.00, 7.00)	5.00 (3.00, 7.00)	5.00 (3.00, 7.00)
Charlson comorbidity index Median (Q1, Q3)	8.00 (6.00, 10.0)	5.00 (3.00, 6.00)	5.00 (3.00, 7.00)
Full code upon admission	5,536 (90.1%)	48,895 (92.5%)	54,431 (92.3%)
Full code upon discharge	4,836 (78.7%)	43,527 (82.4%)	48,363 (82.0%)
Hypertension	3,605 (58.7%)	32,061 (60.7%)	35,666 (60.5%)
Heart Failure	1,443 (23.5%)	15,105 (28.6%)	16,548 (28.1%)
Asthma	180 (2.9%)	3,184 (6.0%)	3,364 (5.7%)
COPD	1,535 (25.0%)	12,854 (24.3%)	14,389 (24.4%)
CKD	564 (9.2%)	6,299 (11.9%)	6,863 (11.6%)

Abbreviations: CKD, chronic kidney disease; COPD, chronic obstructive pulmonary disease; Q1, first quartile; Q3, third quartile; SOFA, Sequential Organ Failure Assessment Score.

### 3.1 Propensity to receive treatment

**FIGURE 2:**
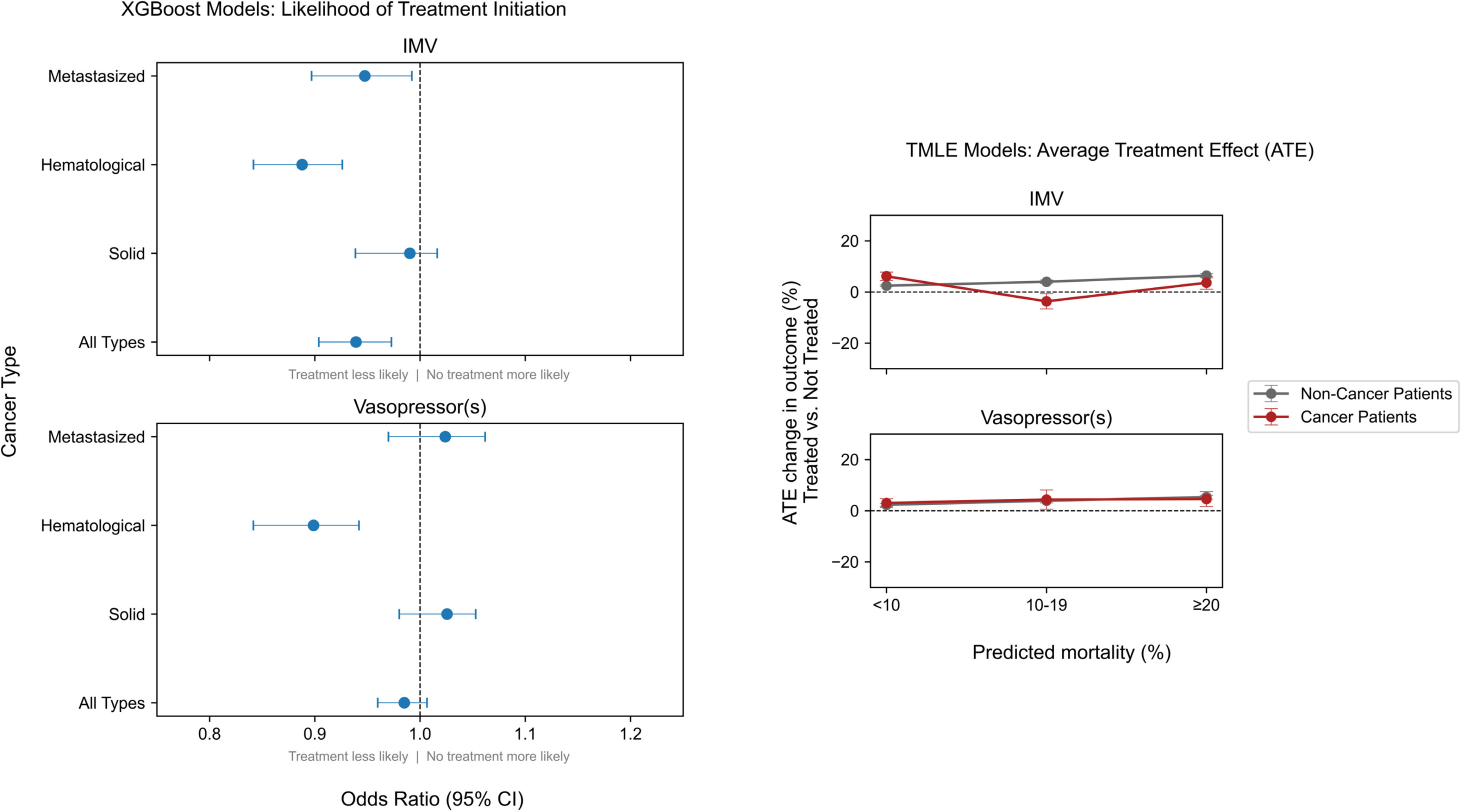
Results of the XGBoost (left panel) and TMLE models (right panel) for the odds of a cancer patient receiving invasive treatments and the average treatment effects (ATE) for change in in‐hospital mortality over predicted mortality categories compared to a non‐cancer patient. CI, confidence interval; IMV, invasive mechanical ventilation; TMLE, targeted maximum likelihood estimation.

We estimated the propensity to receive an intervention by a XGBoost model. Patients with metastasized or hematological cancer were less likely to be treated with IMV (OR 0.95, 95% CI 0.90–0.99, and OR 0.89, 95% CI 0.84–0.93, respectively) (see Figure 2, left panel, and Supplementary Table 5A). This inequality was only observed for hematological cancer patients regarding treatment with vasopressors (OR 0.90, 95% CI 0.84–0.94).

### 3.2 Causal effect on outcomes

All strata had non‐null frequencies in receiving one of the interventions, thus fulfilling the positivity assumption (see Supplementary Tables 6A,B). The negative control outcome of death or discharge at an even hour versus at an odd hour showed no deviation from the anticipated random effect (see Supplementary Figure 3 and Supplementary Tables 5B,C) supporting the validity of our TMLE models.

In‐hospital mortality was comparable between cancer and non‐cancer patients for both interventions and across all PMP. Both IMV and vasopressors slightly increased the probability of in‐hospital mortality instead of reducing it, no matter if non‐cancer patients (IMV PMP 0%–100%, ATE 4%, 95% CI 4%–4%, and vasopressors PMP 0%–100%, ATE 5%, 95% CI 5%–6%, respectively), or cancer patients (IMV PMP 0%–100%, ATE 2%, 95% CI 1%–4%, and vasopressors PMP 0%–100%, ATE 4%, 95% CI 2%–5%, respectively) were analyzed (see Supplementary Table 5). Yet between predicted PMP strata for IMV, a difference in ATE was observed, with a statistically significant improvement in mortality for cancer patients with an intermediate predicted mortality risk of 10%–19%.

**FIGURE 3:**
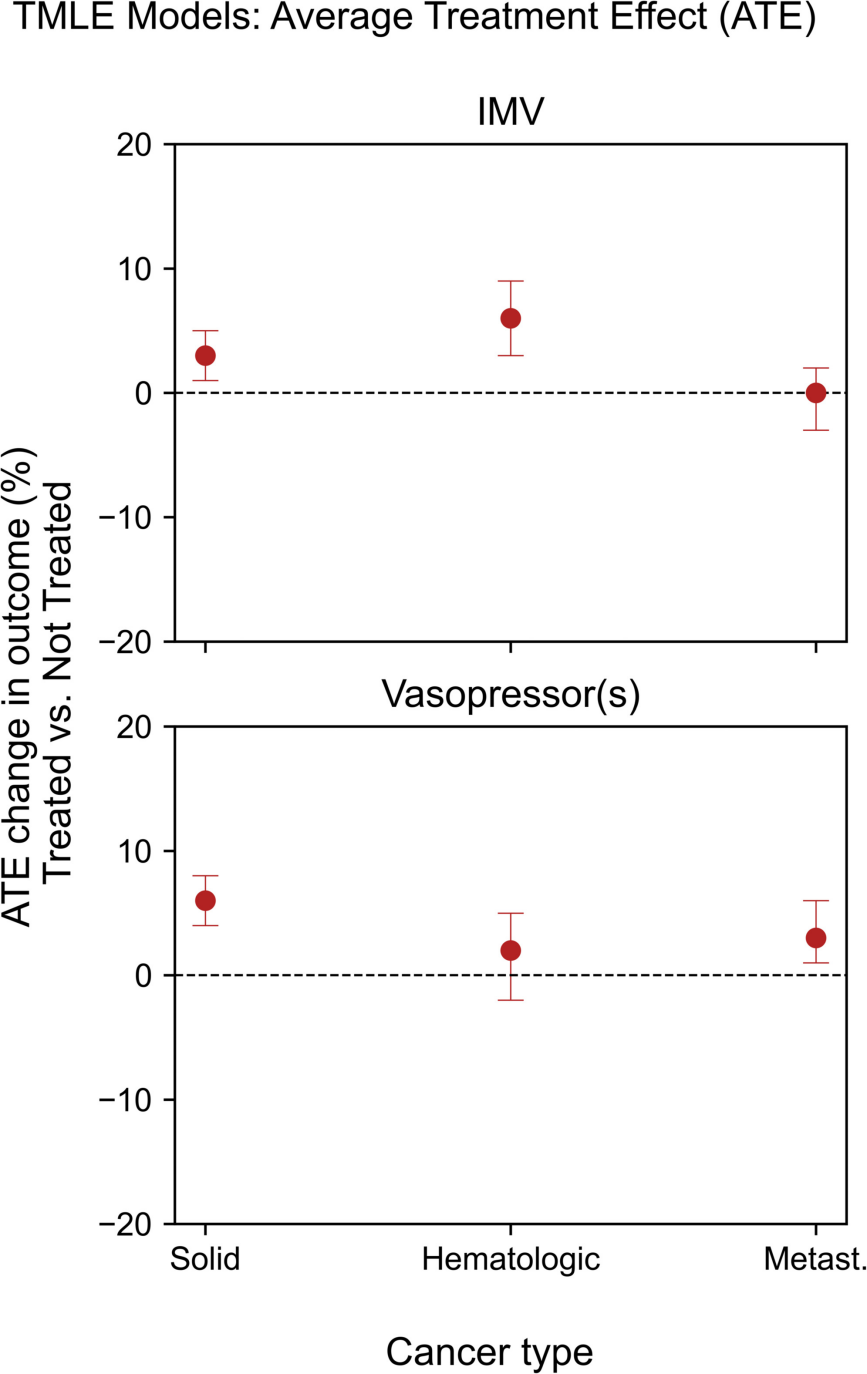
TMLE derived ATEs for change in in‐hospital mortality for different cancer types compared to non‐cancer patients. ATE, average treatment effect; IMV, invasive mechanical ventilation; TMLE, targeted maximum likelihood estimation.

28‐hospital free days were reduced by the interventions in both cancer and non‐cancer patients in all PMP strata. When analyzing the three groups of cancer types separately, we observed the same outcomes, albeit with more variation due to the reduced sample size (see Figure 3 and Supplementary Table 5C). In a sensitivity analysis, we considered MIMIC‐IV and eICU‐CRD separately. All results were similar across databases for both the XGBoost models and the TMLE models (see supplementary Figures 4A, B, and 5).

We wanted to investigate if the in‐hospital interventions were also affecting post‐discharge outcomes. As MIMIC‐IV provides 1 year mortality data, we analyzed this data separately with a TMLE model setting without any treatment to estimate the baseline mortality. Baseline 1 year mortality was quite heterogeneous, with 30% for non‐cancer patients, 48% for solid cancer patients, 58% for hematological cancer patients, and 77% for metastasized cancer patients (overall 62%, see Table 2). Despite these differences, the ATE was very similar between the groups when an intervention was added to the TMLE models.

**TABLE 2:** TMLE derived casual average treatment effects for the change in 1 year mortality from baseline mortality for different cancer types. MIMIC‐IV only.

Treatment	Baseline mortality	Average treatment effect (95% CI)		*N*
		IMV	Vasopressors	
Any cancer	62% (61%–64%)	−3% (−6% to 1%)	−1% (−4% to 2%)	3,875
Solid	48% (45%–51%)	1% (−6% to 7%)	3% (−3% to 1%)	1,430
Hematological	58% (55%–62%)	−2% (−10% to 6%)	**−14% (−21% to** −**8%**)	910
Metastasized	77% (75%–80%)	−2% (−7% to 4%)	−2% (−7 to 4%)	1,535

*Note*: Results not crossing the null are in bold face.

Abbreviation: CI, confidence interval; IMV, invasive mechanical ventilation.

## 4 DISCUSSION

In this observational study of over 65,000 patients, we analyzed data from the MIMIC‐IV and eICU‐CRD databases to dissect treatment patterns between patients with and without cancer and their outcomes. Even though cancer patients showed a significantly higher rate of in‐hospital mortality (30.3%) compared to non‐cancer patients (14.5%), treatment patterns between cancer and non‐cancer patients did not differ significantly, except for a reduced tendency for hematologic patients to receive IMV or vasopressors. Additionally, hospital mortality rates for treated patients showed no significant difference, which is in line with the existing literature showing that the survival of cancer patients receiving critical care is more dependent on age, comorbidities, severity of acute organ dysfunctions, and local practice patterns than on cancer diagnosis per se.^8^, ^31^, ^32^, ^33^

One finding of our study is that neither vasopressors nor IMV on average improved in‐hospital mortality for the whole treated patient population. Overall, invasive therapies seemed to result in higher average in‐hospital mortality rates. This might be explained by a complex relationship between sepsis severity, cancer subtypes, and treatment response. Average treatment effects between cancer and non‐cancer patients were similar and showed no benefit of IMV or vasopressors on both in‐hospital and 1‐year mortality. An exception is the use of vasopressors in the subset of hematologic patients, which reduced 1‐year mortality by an average of 14% (95% CI −21% to −8%). This could potentially be due to a large proportion of acute treatment‐related side effects such as neutropenia, which can successfully be bridged before the immune system recovers.

This study's strengths lie in its large case numbers from more than 200 communal and academic hospitals in the United States, and its coherent use of causal inference methodology^10^, ^11^ to dissect treatment effects in cancer patients with sepsis. Even though restrictions of the retrospective nature remain, this novel approach can provide a framework to obtain novel insights into a patient subgroup that is increasingly represented in ICU admissions. We acknowledge that despite using a thorough causal framework and a negative control outcome, we cannot exclude that our findings are affected by residual, unmeasured confounding.

Yet our findings, which suggest a lack of an ATE of these invasive therapies, are not without precedents, as various randomized controlled trials failed to find differences or benefits for common ICU interventions, as well. This includes fluid resuscitation strategies,^34^, ^35^ delirium management,^36^ early total parenteral nutrition,^37^, ^38^ ECMO^39^, ^40^ or tight glycemic control.^41^, ^42^ Conventionally, this is attributed to a lack of statistical power. However, the interventions themselves may not be beneficial to subsets of the included study populations. Nonetheless, the lack of a signal by ATE does not preclude potential beneficial effects in distinct subgroups of patients or for individual patients. While very narrow randomized controlled trials with a specific patient population could answer such questions, it would be ethically unjustifiable to randomize patients based on a single retrospective study. Here, similar analyses using other and more granular databases may help to delineate individual treatment effects to identify the subgroup of patients where the benefits of a treatment outweigh the adverse effects. Such variables could be time‐resolved organ dysfunction scores, inflammatory biomarkers (e.g., procalcitonin, IL‐6, CRP, etc.), baseline frailty, and oncologic details (e.g., cancer stage, treatment intent) to enable better stratification and identify subgroups of patients who may truly benefit from ICU interventions.

## 5 LIMITATIONS

Our study has some important limitations. First, the study's reliance on US data only may limit its generalizability to other settings with different admission policies or treatment protocols. Especially, local culture can affect ICU admission policy strongly, impacting the ICU population studied and changing the findings. Meanwhile, these patient selection policies are hard to derive from observational data. Second, the eICU‐CRD dataset does not include survival data beyond the hospital stay; therefore, we cannot infer any long‐term outcomes for these patients. Third, we lacked cancer‐specific details, such as cancer subtypes, TNM stages, treatment regimens, or tumor genomics. However, we are unaware of other datasets that could provide the granularity required to answer such questions. This gap, coupled with inadequate case numbers, hindered our ability to conduct detailed tumor‐specific analyses and further dissect patient groups. Finally, our study has an intrinsic potential for biases due to its observational design, including unmeasured confounders. We aimed to mitigate these effects by using a rigorous target trial design with a negative control outcome.

## 6 CONCLUSION

In conclusion, our findings suggest no survival benefit for invasive therapies in sepsis patients with cancer and are a spotlight on the need to further study patient outcomes at subgroup levels. Furthermore, it highlights the importance of careful selection of patients to whom invasive therapies are administered, regardless of a potential cancer diagnosis.

By making our models and approach publicly available, we hope that others will utilize it for other hard‐to‐treat patient groups as well, thereby helping to better understand who benefits from invasive therapies and who does not. Future research should focus on individual treatment effects, subgroup analyses, and functional status to identify patients benefiting from specific treatments. These efforts are essential for refining treatment strategies and improving outcomes, particularly in an aging society with increased financial constraints and growing technological possibilities.

## AUTHOR CONTRIBUTIONS

**João Matos:** Writing – review and editing; visualization; methodology; data curation; conceptualization; funding acquisition. **Tristan Struja:** Conceptualization; methodology; writing – original draft; data curation; funding acquisition. **Naira Link Woite:** Writing – review and editing; visualization. **David Restrepo:** Data curation; software; formal analysis. **Andre Kurepa Waschka:** Methodology; project administration; supervision. **Leo A. Celi:** Supervision; conceptualization. **Christopher M. Sauer:** Conceptualization; methodology; supervision; project administration; funding acquisition.

## FUNDING INFORMATION

CMS is supported by the German Research Foundation funded UMEA Clinician Scientist Program (FU356/12‐2). TS (Swiss National Science Foundation, P400PM_194497 / 1). JM (Fulbright / FLAD Grant, Portugal, AY 2022/2023). LAC (NIBIB, R01 EB001659).

The funding organizations had no role in the design and conduct of the study; collection, management, analysis, and interpretation of the data; preparation, review, or approval of the manuscript; and decision to submit the manuscript for publication.

## CONFLICT OF INTEREST STATEMENT

C.M. Sauer does unrelated freelancing work for Pacmed B.V., which owns an AI tool for ICU discharge decision support. All other authors declare no conflicts of interest relevant to this work.

## ETHICS STATEMENT

The data in MIMIC‐IV has been previously de‐identified, and the institutional review boards of the Massachusetts Institute of Technology (No. 0403000206) and BIDMC (2001‐P‐001699/14) both approved the use of the database for research and waived the need for informed consent. The eICU‐CRD database is a publicly available multi‐center database sourced from the Philips Healthcare eICU Telehealth Program and contains information from over 200,000 admissions to ICUs monitored by eICU programs across the United States. Use of the eICU database is exempt from institutional review board approval due to the retrospective design, lack of direct patient intervention, and security schema, for which the re‐identification risk was certified as meeting safe harbor standards by an independent privacy expert (Privacert, Cambridge, MA, Health Insurance Portability and Accountability Act Certification No. 1031219‐2).

## Supporting information


**Supplementary Table 1.** Summary of the target trial protocol.
**Supplementary Table 2**. Classification and distribution of cancer types for the merged.
**Supplementary Table 3**. Baseline information by dataset.
**Supplementary Table 4**. List of confounders included into the models.
**Supplementary Table 5A**. Exact values reported in Figure 2.
**Supplementary Table 5B**. Exact values for all results of TMLE models reported in Figure 2.
**Supplementary Table 5C**. Exact values for all results of TMLE models reported in Figure 3.
**Supplementary Table 6A**. Frequency of Treatment across predicted mortality ranges stratified by in‐hospital mortality and cancer status.
**Supplementary Table 6B**. Frequency of treatment and cancer type stratified by in‐hospital mortality.
**Supplementary Figure 1**. Causal direct acyclic graph.
**Supplementary Figure 2**. Sequence of analytical methods used in this study.
**Supplementary Figure 3**. TMLE derived ATEs for negative control outcome with death or discharge at odd versus even hour.
**Supplementary Figure 4A**. Results of the XGBoost models (5‐fold and 20 replications each) for the odds of a cancer patient receiving.
**Supplementary Figure 4B**. Results of the XGBoost models (5‐fold and 20 replications each) for the odds of a cancer patient receiving.
**Supplementary Figure 5**. TMLE derived ATEs for change in in‐hospital mortality for different Cancer Types. Panel A (left) MIMIC‐IV.

## Data Availability

The data underlying this study are reported with the identifier doi.org/10.1093/jamia/ocx084 (MIMIC‐IV) and with the identifier doi.org/10.1038/sdata.2018.178 (eICU‐CRD). Both databases are publicly available in PhysioNet (https://physionet.org/). The code that produces the results in this manuscript can be accessed at https://github.com/joamats/mit-tmle-cancer, which includes detailed instructions for running the code. Further information is available from the corresponding author upon request.
