# Supplementary material for: A causal inference framework to compare the effectiveness of life‐sustaining ICU therapies—using the example of cancer patients with sepsis
Source: Int J Cancer. 2025 Sep 8;158(3):707–15. doi: 10.1002/ijc.70138 (PMC12670347; doi:10.1002/ijc.70138)
Supplement: Supplementary file 1 — Supplementary Table 1. Summary of the target trial protocol. Supplementary Table 2. Classification and distribution of cancer types for the merged. Supplementary Table 3. Baseline information by dataset. Supplementary Table 4. List of confounders included into the models. Supplementary Table 5A. Exact values reported in Figure 2. Supplementary Table 5B. Exact values for all results of TMLE models reported in Figure 2. Supplementary Table 5C. Exact values for all results of TMLE models reported in Figure 3. Supplementary Table 6A. Frequency of Treatment across predicted mortality ranges stratified by in‐hospital mortality and cancer status. Supplementary Table 6B. Frequency of treatment and cancer type stratified by in‐hospital mortality. Supplementary Figure 1. Causal direct acyclic graph. Supplementary Figure 2. Sequence of analytical methods used in this study. Supplementary Figure 3. TMLE derived ATEs for negative control outcome with death or discharge at odd versus even hour. Supplementary Figure 4A. Results of the XGBoost models (5‐fold and 20 replications each) for the odds of a cancer patient receiving. Supplementary Figure 4B. Results of the XGBoost models (5‐fold and 20 replications each) for the odds of a cancer patient receiving. Supplementary Figure 5. TMLE derived ATEs for change in in‐hospital mortality for different Cancer Types. Panel A (left) MIMIC‐IV. [file IJC-158-707-s001.docx]

A Causal Inference Framework to compare the effectiveness of life-sustaining ICU therapies - using the example of cancer patients with sepsis

**Running Title:** TMLE Cancer

**Author List**

João Matos, M.Sc. 1, 2*, Tristan Struja, M.D., M.Sc, M.P.H. 2, 3*, Naira Link Woite, M.D., M.P.H., 2, David Restrepo, M.Sc., 2, 4, Andre Kurepa Waschka, Ph.D., 5, Leo A. Celi, M.D., M.Sc., M.P.H., 2, Christopher M. Sauer, M.D. M.P.H. Ph.D. 2, 6

* shared first authors

**Author Affiliations**

1. Faculty of Engineering of University of Porto, Porto, Portugal
2. Laboratory for Computational Physiology, Institute for Medical Engineering and Science, Massachusetts Institute of Technology, Cambridge, MA, USA
3. Medical University Clinic, Kantonsspital Aarau, Aarau, and Spital Muri, Muri, Switzerland
4. Telematics Department, University of Cauca, Popayan, Colombia
5. Mercer University, Macon, GA, and Elon University, Elon NC, USA
6. Department of Hematology & Stem Cell Transplantation, West German Cancer Institute, University Hospital Essen, Germany; Institute for Artificial Intelligence in Medicine, University Hospital Essen, Germany

**Corresponding Authors**

Tristan Struja, ORCID 0000-0003-0199-0184, Email: [tstruja@mit.edu](mailto:tstruja@mit.edu)

And Christopher M. Sauer, M.D. M.P.H. Ph.D., Email: [sauerc@mit.edu](mailto:sauerc@mit.edu)

**Supplemental Online Material**

### **Page 3 - Supplementary Table 1.** Summary of the target trial protocol

### **Page 4 - Supplementary Table 2.** Classification and distribution of cancer types for the merged MIMIC-IV and eICU-CRD cohorts based on ICD-10 codes

### **Page 5 - Supplementary Table 3.** Baseline information by dataset

### **Page 7 - Supplementary Table 4.** List of confounders included into the models.

### **Page 9 - Supplementary Table 5A.** Exact values reported in Figure 2.

### **Page 10 - Supplementary Table 5B.** Exact values for all results of TMLE models reported in Figure 2.

### **Page 11 - Supplementary Table 5C.** Exact values for all results of TMLE models reported in Figure 3.

### **Page 12 - Supplementary Table 6A.** Frequency of Treatment across predicted mortality ranges stratified by in-hospital mortality and cancer status checking for violations of TMLE’s positivity assumption. Cells unevenly populated must be taken with caution when reporting conclusions

### **Page 12 - Supplementary Table 6B.** Frequency of treatment and cancer type stratified by in-hospital mortality.

**Page 13 - Supplementary Figure 1.** Causal direct acyclic graph.

**Page 14 - Supplementary Figure 2.** Sequence of analytical methods used in this study.

**Page 15 - Supplementary Figure 3.** TMLE derived ATEs for negative control outcome with death or discharge at odd versus even hour.

**Page 16 - Supplementary Figure 4A.** Results of the XGBoost models (5-fold and 20 replications each) for the odds of a cancer patient receiving an invasive treatment and the TMLE models for average treatment effect (ATE) for in-hospital mortality only compared to a non-cancer patient. MIMIC-IV only.

**Page 17 - Supplementary Figure 4B.** Results of the XGBoost models (5-fold and 20 replications each) for the odds of a cancer patient receiving an invasive treatment and the TMLE models for average treatment effect (ATE) for in-hospital mortality compared to a non-cancer patient. eICU-CRD only.

### **Page 18 - Supplementary Figure 5.** TMLE derived ATEs for change in in-hospital mortality for different Cancer Types. Panel A (left) MIMIC-IV, and Panel B (right) eICU-CRD.

This supplemental material has been provided by the authors to give readers additional information about their work.

### **SUPPLEMENTARY MATERIAL**

### **Supplementary Table 1.** Summary of the target trial protocol

| **Protocol component** | **Description** |
| --- | --- |
| **Eligibility criteria** | Patients admitted to the ICU from 2008-2019 (MIMIC-IV) and 2014-2015 (eICU-CRD) for the first time during their hospital stay, fulfilling the Sepsis-3 criteria and aged 18 years or older. Length of ICU stay of at least 1 day. Furthermore, patients had to have an oncological ICD-10 code registered for the relevant stay (Suppl. Table 2) |
| **Treatment strategies** | Initiation of invasive mechanical ventilation versus no invasive ventilation within 24 hours of admission until discharge or death. Initiation of vasopressors versus no use of vasopressors within 24 hours of admission until discharge or death. |
| **Assignment procedures** | Patients were eligible to receive the treatment within the first 24 hours of their ICU stay. If treatment was started after this eligibility period, the patient was retained in the control group to avoid immortal time bias. Patients and providers will be aware of the strategy to which they have been assigned. |
| **Follow-up period** | Starts at randomization/ after treatment initiation within the initial 24 hours of admission and ends at discharge or death whichever occurs first. |
| **Outcomes** | Primary outcome: in-hospital mortality, including discharge to hospice care. Secondary outcomes: 28-hospital free days, 1-year mortality (MIMIC-IV patients only, as follow-up data is not available in eICU-CRD).  Negative control outcome: timing of discharge or death at an odd versus an even hour. |
| **Causal contrasts of interest** | Intention-to-treat effect. |
| **Analysis plan** | Intention-to-treat effect estimated via comparison of patients receiving the treatment compared to those not receiving the treatment within the first 24 hours of admission, adjusted for the extensive list of prespecified confounders. Estimation of average treatment effects using TMLE. |

### **Supplementary Table 2.** Classification and distribution of cancer types for the merged MIMIC-IV and eICU-CRD cohorts based on ICD-10 codes

| **Cancer** | **Terminology** | **ICD-10 Codes** | **Distribution in the cohort**  **(N=6,145)** |
| --- | --- | --- | --- |
| Cancer Categories | Solid, excluding metastatic disease | C00 - C43, C4A,  C45 - C76, C80 | 2,798 (45.5%) |
|  | Hematological | C81 - C86, C88,  C90 - C96 | 1,427 (23.2%) |
|  | Metastatic | C77 - C7, C7B | 1,920 (31.2%) |
| Cancer Types* | Colon and Rectal | C17 - C21 | 430 (7.0%) |
|  | Liver and Intrahepatic Bile Duct | C22 | 362 (5.9%) |
|  | Pancreatic | C25 | 303 (4.9%) |
|  | Other digestive | C0X, C14 - 16 | 365 (5.9%) |
|  | Lung | C34 | 1,139 (18.5%) |
|  | Other respiratory | C30 - 33, C37 - 39 | 67 (1.1%) |
|  | Other mesothelial | C40 - 41, C45 - 49 | 91 (1.5%) |
|  | Melanoma | C43 | 55 (0.9%) |
|  | Breast | C50 | 175 (2.8%) |
|  | Female genital | C53 - C58 | 201 (3.3%) |
|  | Male genital | C61 - 63 | 341 (5.5%) |
|  | Renal and Urinary | C64 - C68 | 339 (5.5%) |
|  | Central nervous system | C69 - 72 | 304 (4.9%) |
|  | Endocrine | C73 - 75, C7B | 53 (0.9%) |
|  | Lymphomas | C81 - C86, C88 | 575 (9.4%) |
|  | Leukemia | C90 - C95 | 860 (14.0%) |
|  | Others | C76 - 80 | 1,958 (31.9%) |
|  | **Total** |  | **7,618** |

**Legend**: *Multiple primary malignancies possible why total is higher than number of patients; ICD-10 codes, International Classification of Diseases, Tenth Revision;

### **Supplementary Table 3.** Baseline information by dataset

|  | **MIMIC-IV (N=23,619)** | **eICU-CRD (N=35,369)** | **Overall (N=58,988)** |
| --- | --- | --- | --- |
| **In-hospital Mortality** | 4,650 (19.7%) | 4,870 (13.8%) | 9,520 (16.1%) |
| **Length of stay if died (d)**  Median (Q1, Q3) | 4.54 (2.38, 8.96) | 5.38 (2.63, 10.8) | 4.96 (2.50, 9.84) |
| **Length of stay if survived (d)**  Median (Q1, Q3) | 2.96 (1.75, 5.79) | 6.91 (4.27, 11.7) | 5.33 (2.85, 9.75) |
| **Invasive Mechanic Ventilation** | 11,133 (47.1%) | 16,793 (47.5%) | 27,926 (47.3%) |
| **Renal Replacement Therapy** | 1,762 (7.5%) | 2,683 (7.6%) | 4,445 (7.5%) |
| **Vasopressor(s)** | 10,718 (45.4%) | 14,403 (40.7%) | 25,121 (42.6%) |
| **Cancer Present** | 3,875 (16.4%) | 2,270 (6.4%) | 6,145 (10.4%) |
| **Age overall (yrs)**  Median (Q1, Q3) | 67.0 (55.0, 77.0) | 67.0 (55.0, 78.0) | 67.0 (55.0, 78.0) |
| **Sex (Female)** | 9,924 (42.0%) | 16,982 (48.0%) | 26,906 (45.6%) |
| **Race** |  |  |  |
| Asian | 664 (2.8%) | 476 (1.3%) | 1,140 (1.9%) |
| Black | 2,045 (8.7%) | 3,891 (11.0%) | 5,936 (10.1%) |
| Hispanic | 816 (3.5%) | 1,656 (4.7%) | 2,472 (4.2%) |
| Other | 4,039 (17.1%) | 1,964 (5.6%) | 6,003 (10.2%) |
| White | 16,055 (68.0%) | 27,382 (77.4%) | 43,437 (73.6%) |
| **SOFA** Median (Q1, Q3) | 5.00 (3.00, 8.00) | 4.00 (2.00, 6.00) | 5.00 (3.00, 7.00) |
| **Charlson Comorbidity Index**  Median (Q1, Q3) | 6.00 (4.00, 8.00) | 4.00 (2.00, 6.00) | 5.00 (3.00, 7.00) |
| **Full Code upon Admission** | 22,289 (94.4%) | 32,142 (90.9%) | 54,431 (92.3%) |
| **Full Code upon Discharge** | 21,633 (91.6%) | 26,730 (75.6%) | 48,363 (82.0%) |
| **Hypertension** | 15,488 (65.6%) | 20,178 (57.1%) | 35,666 (60.5%) |
| **Heart Failure** | 7,921 (33.5%) | 8,627 (24.4%) | 16,548 (28.1%) |
| **Asthma** | 302 (1.3%) | 3,062 (8.7%) | 3,364 (5.7%) |
| **COPD** | 5,563 (23.6%) | 8,826 (25.0%) | 14,389 (24.4%) |
| **CKD** | 2,726 (11.5%) | 4,137 (11.7%) | 6,863 (11.6%) |
| **Hematological Cancer** | 910 (3.9%) | 517 (1.5%) | 1,427 (2.4%) |
| **Metastasized Cancer** | 1,535 (6.5%) | 385 (1.1%) | 1,920 (3.3%) |
| **Solid Cancer** | 1,430 (6.1%) | 1,368 (3.9%) | 2,798 (4.7%) |
| **Cancer Types*** |  |  |  |
| Colon and Rectal (combined) | 202 (0.9%) | 228 (0.6%) | 430 (0.7%) |
| Liver and intrahepatic Bile Duct | 326 (1.4%) | 36 (0.1%) | 362 (0.6%) |
| Pancreatic | 237 (1.0%) | 66 (0.2%) | 303 (0.5%) |
| Other digestive | 226 (1.0%) | 139 (0.4%) | 365 (0.6%) |
| Other mesothelial | 52 (0.2%) | 39 (0.1%) | 91 (0.2%) |
| Lung (including bronchus) | 606 (2.6%) | 533 (1.5%) | 1,139 (1.9%) |
| Other respiratory | 39 (0.2%) | 28 (0.1%) | 67 (0.1%) |
| Melanoma | 30 (0.1%) | 25 (0.1%) | 55 (0.1%) |
| Breast | 78 (0.3%) | 97 (0.3%) | 175 (0.3%) |
| Female genital | 102 (0.4%) | 99 (0.3%) | 201 (0.3%) |
| Male genital | 200 (0.8%) | 141 (0.4%) | 341 (0.6%) |
| Renal and urinary | 214 (0.9%) | 125 (0.4%) | 339 (0.6%) |
| Central nervous system | 125 (0.5%) | 179 (0.5%) | 304 (0.5%) |
| Endocrine | 44 (0.2%) | 9 (0.0%) | 53 (0.1%) |
| Lymphomas | 397 (1.7%) | 178 (0.5%) | 575 (1.0%) |
| Leukemia | 524 (2.2%) | 336 (1.0%) | 860 (1.5%) |
| Others | 1,558 (6.6%) | 400 (1.1%) | 1,958 (3.3%) |

**Legend**: *Multiple primary malignancies possible why total is higher than number of patients; Q1, First Quartile; Q3, third quartile; SOFA, Sequential Organ Failure Assessment Score; COPD, Chronic Obstructive Pulmonary Disease; CKD, Chronic Kidney Disease

### **Supplementary Table 4.** List of confounders included into the models.

| **Confounder** | **Details** | **Confounder** | **Details** |
| --- | --- | --- | --- |
| Age | N/A | Respiration rate | Mean first 24h |
| Sex | N/A | Mean blood pressure | Mean first 24h |
| Ethnicity | N/A | Heart rate | Mean first 24h |
| Year | 2 yearly bins provided by dataset | Temperature | Mean first 24h |
| Elective admission yes/no | N/A | Spo2 | Mean first 24h |
| Major surgery yes/no | N/A | pO2 | Minimum first 24h |
| Full code at admission yes/no | N/A | pCO2 | Maximum first 24h |
| Full code at discharge yes/no | N/A | pH | Minimum first 24h |
| Illness severity, probability of dying | OASIS (MIMIC) and APACHE (eICU) at admission | Lactate | Maximum first 24h |
| SOFA score first 24 hours | N/A | Glucose | Maximum first 24h |
| SOFA respiration component | N/A | Sodium | Minimum first 24h |
| SOFA coagulation component | N/A | Potassium | Minimum first 24h |
| SOFA liver component | N/A | Cortisol | Minimum first 24h |
| SOFA cardiovascular component | N/A | Hemoglobin | Minimum first 24h |
| SOFA CNS component | N/A | Fibrinogen | Minimum first 24h |
| SOFA renal component | N/A | INR | Maximum first 24h |
| Charlson comorbidity index | N/A | Hypertension yes/no | N/A |
| Hospital anonymous ID | N/A | Heart failure yes/no | N/A |
| Number of beds categorized | as per eICU categorization | COPD yes/no | N/A |
| Teaching hospital yes/no | N/A | Asthma yes/no | N/A |
| Region of hospital | US census regions | Coronary artery disease yes/no | N/A |
| Pneumonia | Site of infection from ICD codes | Chronic kidney disease yes/no | N/A |
| Urinary tract infection | Site of infection from ICD codes | Diabetes | type 1 vs. type 2 |
| Biliary sepsis | Site of infection from ICD codes | Connective disease yes/no | N/A |
| Skin infection | Site of infection from ICD codes |  |  |

**Legend**: N/A, not applicable

### **Supplementary Table 5A.** Exact values reported in Figure 2.

| **XGBoost Model: Likelihood of treatment initiation** | | | |
| --- | --- | --- | --- |
| **Treatment** | **Group** | **Odds Ratio** | **95% CI** |
| **IMV** | Metastasized | **0.95** | **0.90 - 0.99** |
|  | Hematological | **0.89** | **0.84 - 0.93** |
|  | Solid | 0.99 | 0.94 - 1.02 |
|  | All types | **0.94** | **0.90 - 0.97** |
| **Vasopressor(s)** | Metastasized | 1.02 | 0.97 - 1.06 |
|  | Hematological | **0.90** | **0.84 - 0.94** |
|  | Solid | 1.03 | 0.98 - 1.05 |
|  | All types | 0.99 | 0.96 - 1.01 |

**Legend**: CI, Confidence Interval; IMV, Invasive Mechanical Ventilation, results not crossing the null are in **bold face**

### **Supplementary Table 5B.** Exact values for all results of TMLE models reported in Figure 2.

| **TMLE Model** | | | | | | | | | |
| --- | --- | --- | --- | --- | --- | --- | --- | --- | --- |
| **Treatment** | **Group** | **Predicted Mortality  Start - End** | **ATE**  **Mortality** | **95% CI** | **ATE Odd hour** | **95% CI** | **ATE 28-hospital free days** | **95% CI** | **n** |
| **IMV** | Non-cancer Patients | 0% - 9% | **2%** | **2% - 3%** | **1%** | **0% - 3%** | **-2.15** | **-2.25 - -2.04** | 24,260 |
|  |  | 10% - 19% | **4%** | **3% - 5%** | 0% | -1% - 2% | **-2.3** | **-2.48 - -2.12** | 13,141 |
|  |  | 20% - 100% | **6%** | **6% - 7%** | 0% | -1% - 2% | **-2.51** | **-2.70 - -2.32** | 15,442 |
|  |  | 0% - 100% | **4%** | **4% - 4%** | 0% | 0% - 1% | **-2.48** | **-2.56 - -2.41** | 52,843 |
|  | Cancer Patients | 0% - 9% | **6%** | **5% - 8%** | -2% | -7% - 3% | **-1.84** | **-2.25 - -1.43** | 2,572 |
|  |  | 10% - 19% | **-4%** | **-7% - -1%** | 2% | -3% - 8% | -0.52 | -1.19 - 0.15 | 1,559 |
|  |  | 20% - 100% | **4%** | **1% - 6%** | 2% | -3% - 7% | **-2.09** | **-2.68 - -1.50** | 2,014 |
|  |  | 0% - 100% | **2%** | **1% - 4%** | 1% | -1% - 4% | **-1.65** | **-1.95 - -1.35** | 6,145 |
| **Vaso- pressor(s)** | Non-cancer Patients | 0% - 9% | **2%** | **2% - 3%** | 1% | 0% - 3% | **-1.17** | **-1.28 - -1.07** | 24,260 |
|  |  | 10% - 19% | **4%** | **3% - 5%** | 1% | -1% - 2% | **-1.72** | **-1.90 - -1.55** | 13,141 |
|  |  | 20% - 100% | **5%** | **5% - 6%** | -1% | -3% - 0% | **-1.82** | **-1.99 - -1.65** | 15,442 |
|  |  | 0% - 100% | **4%** | **3% - 4%** | 0% | 0% - 1% | **-1.59** | **-1.67 - -1.51** | 52,843 |
|  | Cancer Patients | 0% - 9% | **3%** | **1% - 5%** | 1% | -3% - 5% | **-0.93** | **-1.40 - -0.46** | 2,572 |
|  |  | 10% - 19% | **4%** | **1% - 8%** | 1% | -4% - 7% | **-0.87** | **-1.72 - -1.00** | 1,559 |
|  |  | 20% - 100% | **5%** | **2% - 7%** | -1% | -6% - 4% | **-1.35** | **-1.95 - -0.75** | 2,014 |
|  |  | 0% - 100% | **4%** | **2% - 5%** | 1% | -2% - 4% | **-1.11** | **-1.53 - -0.70** | 6,145 |

**Legend**: CI, Confidence Interval; TMLE, Targeted Maximum Likelihood Estimation; ATE, Average Treatment Effect; IMV, Invasive Mechanical Ventilation; results not crossing the null are in **bold face**

### **Supplementary Table 5C.** Exact values for all results of TMLE models reported in Figure 3.

| **TMLE Model** | | | | | | | | |
| --- | --- | --- | --- | --- | --- | --- | --- | --- |
| **Treatment** | **Group** | **ATE**  **Mortality** | **95% CI** | **ATE Odd hour** | **95% CI** | **ATE 28-hospital free days** | **95% CI** | **n** |
| **IMV** | Solid cancer Patients | **3%** | **1% - 5%** | 0% | -4% - 4% | **-2.19** | **-2.68 - -1.69** | 2,798 |
|  | Hematological cancer Patients | **6%** | **3% - 9%** | 3% | -2% - 8% | **-2.82** | **-3.48 - -2.16** | 1,427 |
|  | Metastasized cancer Patients | 0% | -3% - 2% | 2% | -3% - 7% | -0.47 | -1.04 - 0.09 | 1,920 |
| **Vaso- pressor(s)** | Solid cancer Patients | **6%** | **4% - 8%** | 0% | -4% - 3% | **-1.90** | **-2.39 - -1.42** | 2,798 |
|  | Hematological cancer Patients | 2% | -2% - 5% | 3% | -2% - 8% | 0.00 | -0.82 - 0.82 | 1,427 |
|  | Metastasized cancer Patients | **3%** | **1% - 6%** | 0% | -4% - 5% | **-0.99** | **-1.79 - -0.20** | 1,920 |

**Legend**: CI, Confidence Interval; TMLE, Targeted Maximum Likelihood Estimation; ATE, Average Treatment Effect; IMV, Invasive Mechanical Ventilation; results not crossing the null are in **bold face**

### **Supplementary Table 6A.** Frequency of Treatment across predicted mortality ranges stratified by in-hospital mortality and cancer status checking for violations of TMLE’s positivity assumption. Cells unevenly populated must be taken with caution when reporting conclusions

| **Predicted mortality range** | **< 10 %** | | **10 - 19 %** | | **≥ 20 %** | |
| --- | --- | --- | --- | --- | --- | --- |
|  | **Died (N=1,890)** | **Survived (N=24,942)** | **Died (N=2,162)** | **Survived (N=12,538)** | **Died (N=5,468)** | **Survived (N=11,988)** |
| **Invasive Mechanical Ventilation** |  |  |  |  |  |  |
| in non-cancer patients | 1,386 (73.3%) | 22,874 (91.7%) | 1,691 (78.2%) | 11,450 (91.3%) | 4,579 (83.7%) | 10,863 (90.6%) |
| in cancer patients | 504 (26.7%) | 2,068 (8.3%) | 471 (21.8%) | 1,088 (8.7%) | 889 (16.3%) | 1,125 (9.4%) |
| **Vasopressors** |  |  |  |  |  |  |
| in non-cancer patients | 1,386 (73.3%) | 22,874 (91.7%) | 1,691 (78.2%) | 11,450 (91.3%) | 4,579 (83.7%) | 10,863 (90.6%) |
| in cancer patients | 504 (26.7%) | 2,068 (8.3%) | 471 (21.8%) | 1,088 (8.7%) | 889 (16.3%) | 1,125 (9.4%) |

**Legend**: TMLE, Targeted Maximum Likelihood Estimation

### **Supplementary Table 6B.** Frequency of treatment and cancer type stratified by in-hospital mortality.

| **In-Hospital Outcome** | **Died** | | | **Survived** | | |
| --- | --- | --- | --- | --- | --- | --- |
|  | **Solid (N=627)** | **Hematological (N=463)** | **Metastasized (N=817)** | **Solid (N=2,287)** | **Hematological (N=997)** | **Metastasized (N=1,144)** |
| **Invasive Mechanical Ventilation** | 384 (61.2%) | 271 (58.5%) | 369 (45.2%) | 1,051 (46.0%) | 394 (39.5%) | 406 (35.5%) |
| **Vasopressor(s)** | 407 (64.9%) | 283 (61.1%) | 440 (53.9%) | 959 (41.9%) | 421 (42.2%) | 427 (37.3%) |

**Supplementary Figure 1.** Causal direct acyclic graph.

**
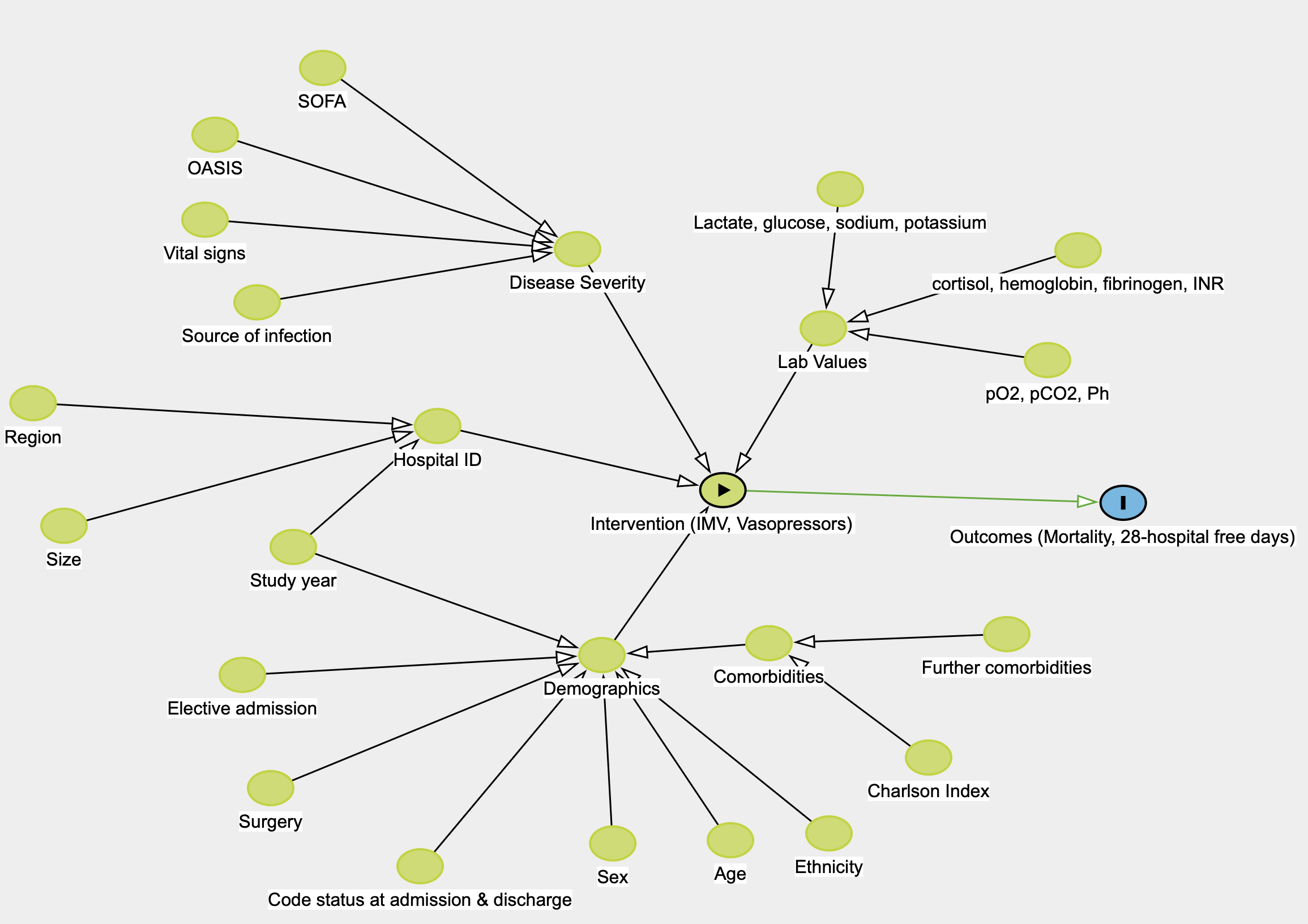

Legend**: Relationships between covariates and outcomes not included for the sake of readability. Created with dagitty.net.

**Supplementary Figure 2.** Sequence of analytical methods used in this study.

*
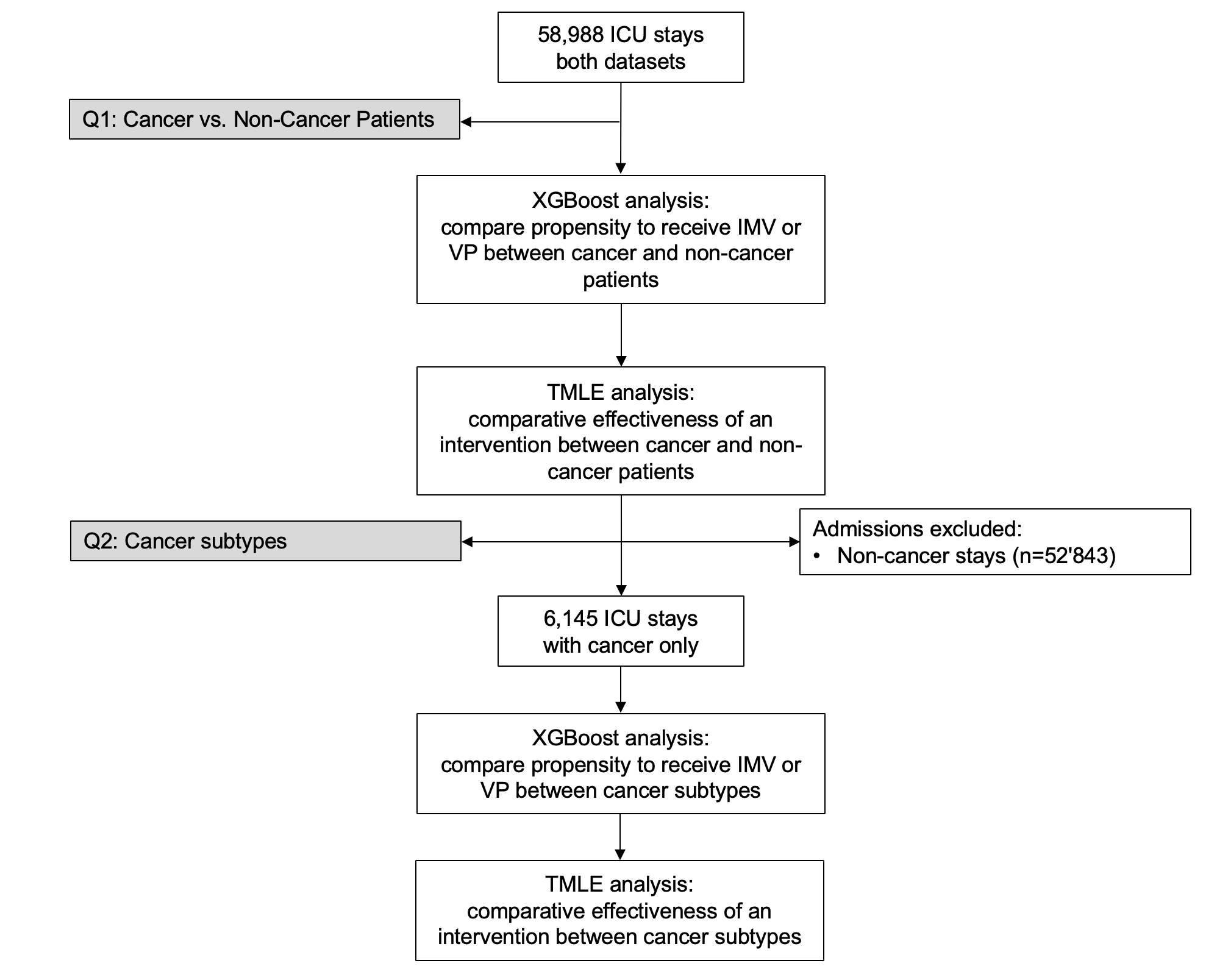
*

**Supplementary Figure 3.** TMLE derived ATEs for negative control outcome with death or discharge at odd versus even hour.

**
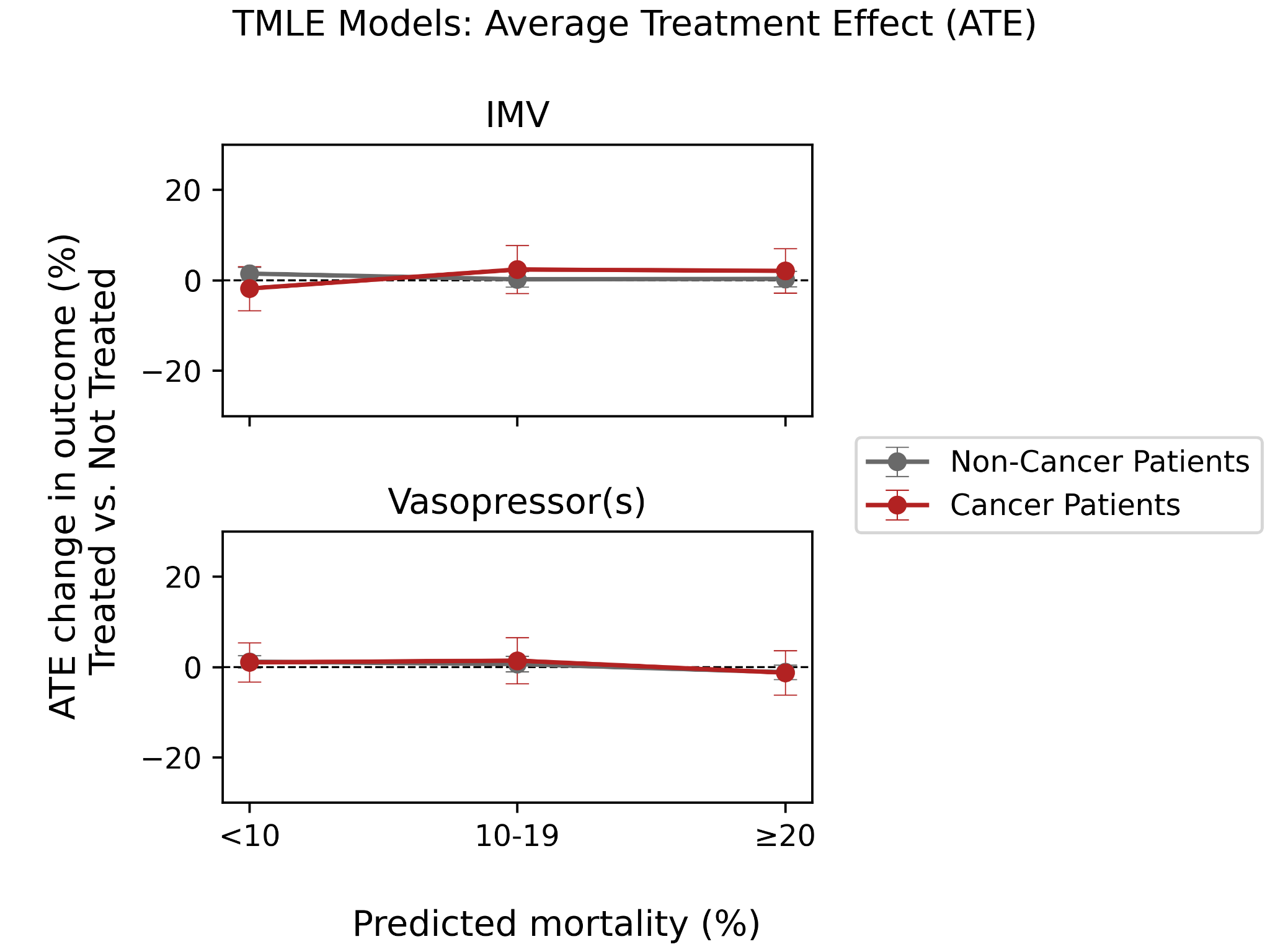
**

### **Legend**: TMLE, Targeted Maximum Likelihood Estimation; IMV, Invasive Mechanical Ventilation

**Supplementary Figure 4A.** Results of the XGBoost models (5-fold and 20 replications each) for the odds of a cancer patient receiving an invasive treatment and the TMLE models for average treatment effect (ATE) for in-hospital mortality only compared to a non-cancer patient. MIMIC-IV only.


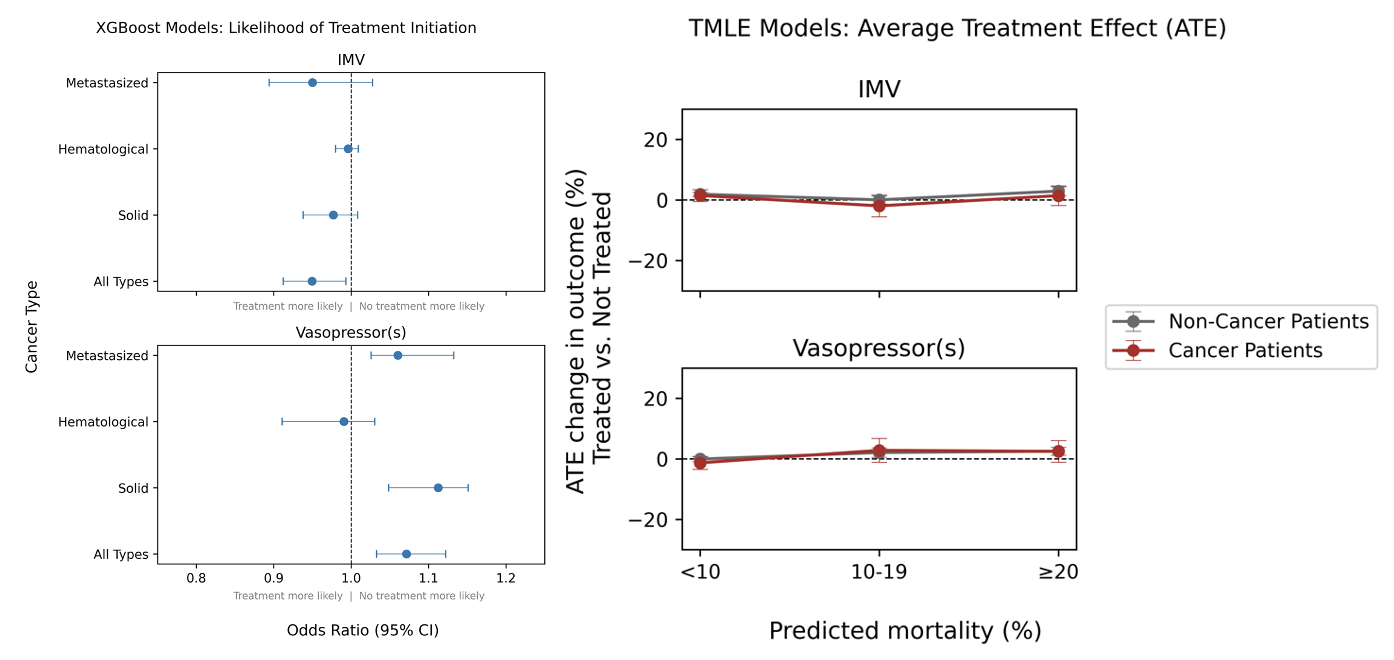


**Legend**: TMLE, Targeted Maximum Likelihood Estimation; CI, Confidence Interval; IMV, Invasive Mechanical Ventilation

**Supplementary Figure 4B.** Results of the XGBoost models (5-fold and 20 replications each) for the odds of a cancer patient receiving an invasive treatment and the TMLE models for average treatment effect (ATE) for in-hospital mortality compared to a non-cancer patient. eICU-CRD only.


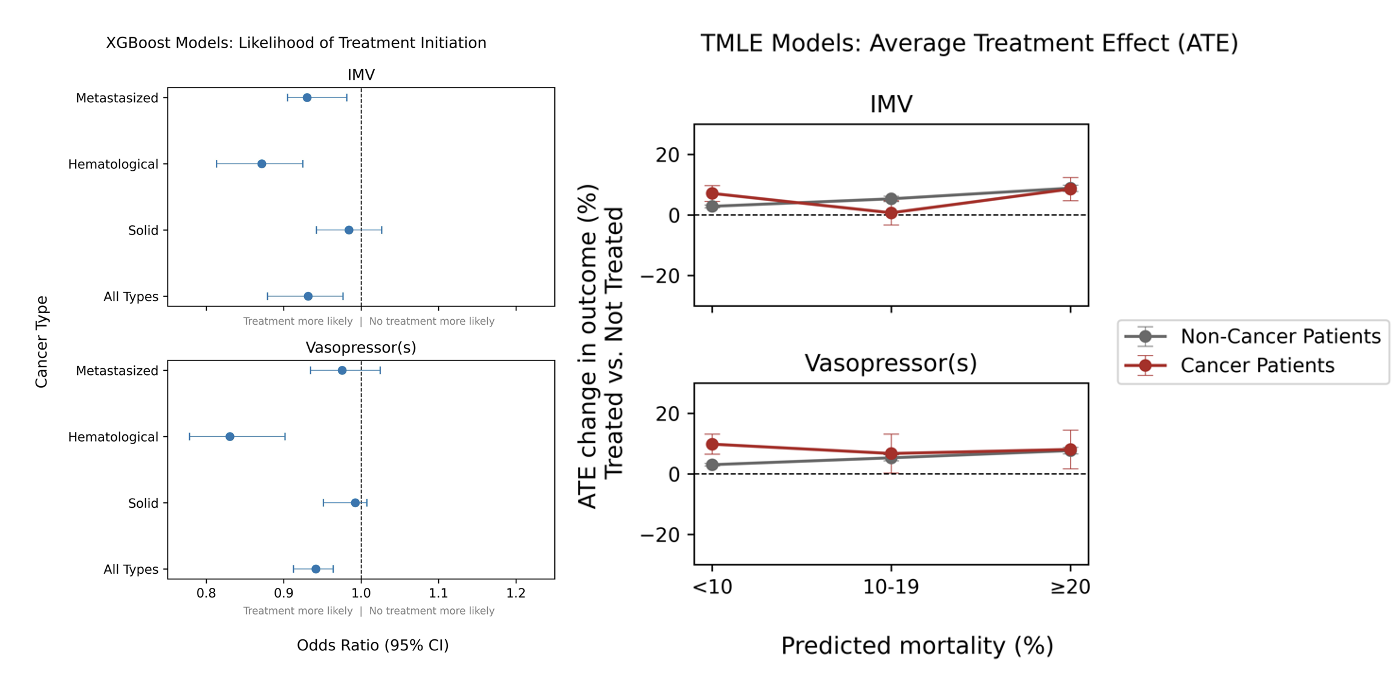


**Legend**: TMLE, Targeted Maximum Likelihood Estimation; CI, Confidence Interval; IMV, Invasive Mechanical Ventilation

### **Supplementary Figure 5.** TMLE derived ATEs for change in in-hospital mortality for different Cancer Types. Panel A (left) MIMIC-IV, and Panel B (right) eICU-CRD.


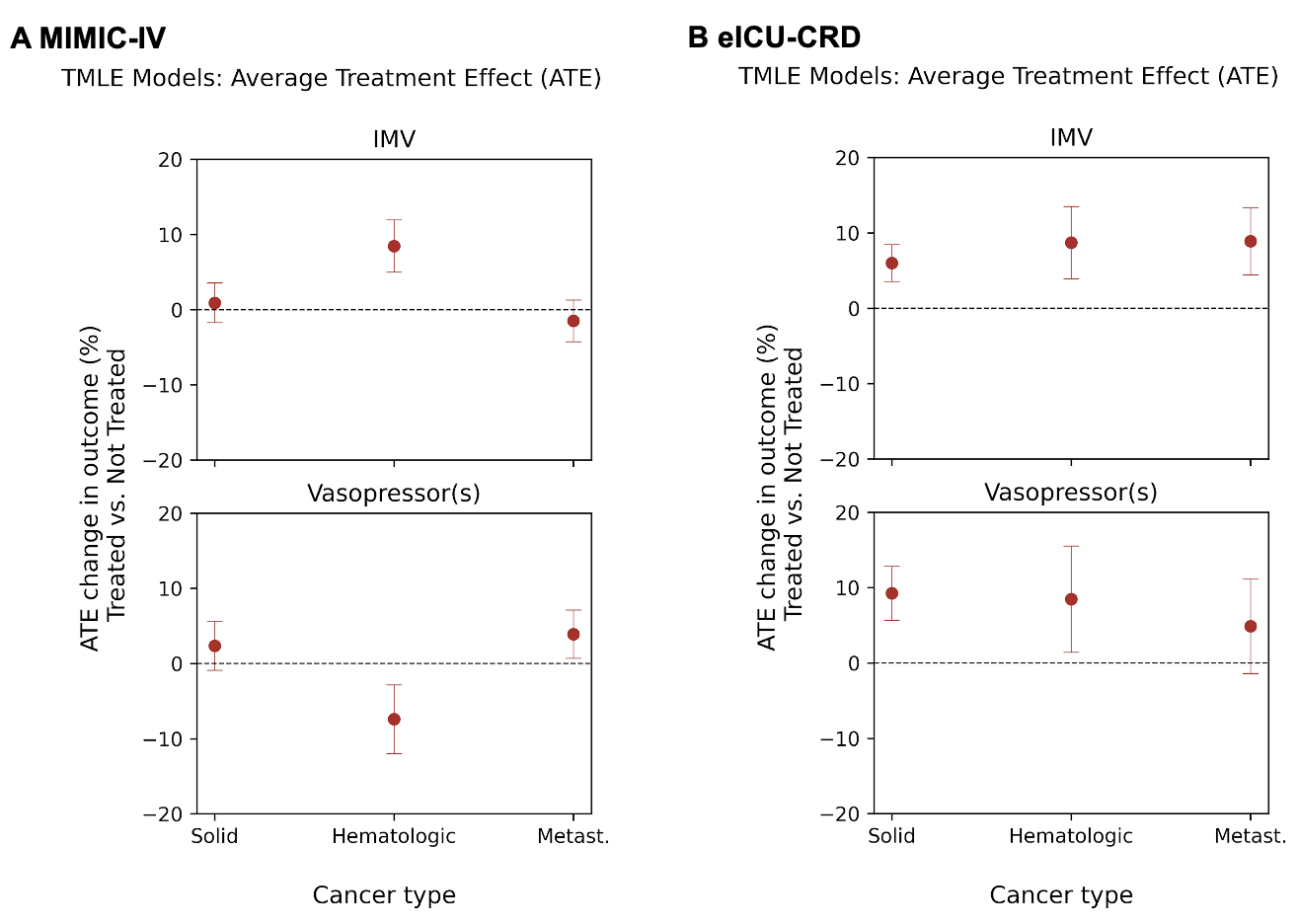


**Legend**: TMLE, Targeted Maximum Likelihood Estimation; CI, Confidence Interval; IMV, Invasive Mechanical Ventilation
